# Does the linear *Sry* transcript function as a ceRNA for miR-138? The sense of antisense

**DOI:** 10.12688/f1000research.3872.2

**Published:** 2014-11-13

**Authors:** Javier Tadeo Granados-Riveron, Guillermo Aquino-Jarquin

**Affiliations:** 1 Laboratorio de Investigación en Genómica, Genética y Bioinformática, Hospital Infantil de México Federico Gómez, Mexico City, 06720, Mexico

## Abstract

Recently, the sex determining region Y ( *Sry*) and the cerebellar degeneration-related protein 1 ( *CDR1as*) RNA transcripts have been described to function as a new class of post-transcriptional regulatory RNAs that behave as circular endogenous RNA sponges for the micro RNAs (miRNAs) miR-138 and miR-7, respectively. A special feature of the *Sry *gene is its ability to generate linear and circular transcripts, both transcribed in the sense orientation. Here we remark that both sense (e.g. *Sry* RNA) and antisense (e.g. *CDR1as*) transcripts could circularize and behave as miRNAs sponges, and importantly, that also protein-coding segments of mRNAs could also assume this role. Thus, it is reasonable to think that the linear *Sry* sense transcript could additionally act as a miRNA sponge, or as an endogenous competing RNA for miR-138.

Crosstalk involving RNA–RNA interactions adds a new dimension to our understanding of complex regulatory networks and offers profound implications for the elucidation of gene function ^1^.

MicroRNAs (miRNAs) are a type of endogenously expressed small regulatory non-protein-coding RNAs that negatively regulate gene expression by base-pairing (with imperfect complementarity) to miRNA response elements (MREs), which are usually located within the 3′-untranslated region (3′-UTR) of target RNA transcripts ^2^. According to their number and location, it has become evident that a biological process may involve multiple miRNAs, and that a given gene may be regulated by more than one miRNA. A recently discovered molecular mechanism, named Competing Endogenous RNA (ceRNA) effect, has highlighted the importance of indirect interactions among transcript RNAs competing for the same pool of miRNAs ^3^. In the case of two ceRNAs having MREs in common, both are regulated by the same set of miRNAs. Multiple classes of non-coding RNAs (lncRNAs) including circular endogenous RNA sponges (circRNAs), pseudogenes, and protein-coding mRNAs function as key ceRNAs and “super-sponges” to regulate the expression of mRNAs in plants and mammalian cells ^4^. The effectiveness of a ceRNA would depend on the number of miRNAs that it can ‘‘absorb’’. This, in turn, would depend on the ceRNA’s accessibility to miRNA molecules, which is influenced by its subcellular localization and its interaction with RNA-binding proteins. Furthermore, the specific cellular context in which the ceRNA is expressed would also impact its overall influence because not all microRNAs are present ubiquitously and at all times ^5^. Aberrant expression of central nodes of such ceRNA networks may cause a disturbance that could contribute to disease pathogenesis ^6^.

Recent bioinformatic and experimental analyses have identified thousands of circRNAs in the mammalian transcriptome, suggesting that circRNAs may in fact represent a new class of ceRNA regulators ^7^. These circRNAs are produced mainly through a type of alternative RNA splicing named ‘back-splicing’, in which a splice donor splices to an upstream acceptor rather than a downstream acceptor ^8^. Recently Guo *et al.* suggested that this would be the way in which most, if not all, cellular circRNAs are generated ^8^.

Recently, Hansen *et al.* and Memczak *et al.* described a new class of post-transcriptional regulatory RNAs that behave as circRNAs in two back-to-back papers published in *Nature* ^9, 10^. In both reports, the authors demonstrated that a ~1.5-kb single-stranded antisense circRNA molecule (human *CDR1as* or *ciRS-7*) containing multiple miR-7 binding sites densely arranged, acts as a natural miRNA sponge, by capturing complexes formed by miR-7/Ago2. Memczak *et al.* observed that human *CDR1as* expression in zebrafish impaired midbrain development, similar to knocking down miR-7 ^9^.

Hansen *et al.* also showed that another circular RNA molecule, transcribed from the mouse *Sry* gene, could also act as an endogenous sponge. They noted that this transcript contains 16 binding sites for miR-138 and demonstrated *in vitro* that the *Sry* circRNA selectively “absorbs” this specific miRNA. Recently, Kartha and Subramanian asserted, based on the report by Hansen *et al.*, that this *Sry* RNA is an antisense circular transcript that functions as a miRNAs sponge ^11^. Although this apparently is a typographical error (antisense instead of sense), it was also referred as such in the original report by Memczak *et al.* in *Nature*. This suggests that the circular *Sry* transcript is, as occurs with the *CDR1as* sponge, an antisense circular RNA. Although it seems obvious that sponges are antisense to the miRNA they bind to, it should not be assumed that all circRNAs are transcripts in an antisense orientation to a protein coding gene, as occurs with *CDR1as*.

Natural antisense transcripts (NATs) are ncRNAs transcribed from the opposite strand of a coding gene and are capable of regulating the expression of their sense gene pair or of several related genes ^12^. Genomic loci that express NATs are highly abundant and sense/antisense (SAS) transcript pairs tend to be co-expressed. The most comprehensive studies predict that in human and mice 40–72% of all transcriptional units show evidence of bi-directional transcription ^13^. To our knowledge, there are no reports of a circRNA whose sequence in sense and antisense orientation, possesses the ability to function as miRNA sponge. A special feature of the *Sry* gene is that it can generate linear as well as circular transcripts depending on the use of alternative promoters (proximal *vs* distal) ^14^. Capel *et al.* reported for the first time that the circular *Sry* RNA is derived from a sense sequence that consists of a single exon. This molecule is formed by the processing of a longer precursor transcript that contains one inverted repeat at each end. This unusual configuration promotes the formation of a stem-loop structure that facilitates the nucleophilic attack of a donor splicing site at the 3′ end to an acceptor site at the 5′ end, which results in its circularization ^14^ ( Figure 1). Thus, it can be asserted that this is, in fact, a circular sense *Sry* mRNA. Although the notion that the *Sry* circRNA is derived from an antisense transcript does not alter the interpretation of the results obtained by Hansen *et al.*, we consider that this distinction is important, because it implies that both sense (e.g. *Sry* RNA) and antisense (e.g. *CDR1as*) transcripts could be circularized and act as RNA sponges, an observation which is not acknowledged by the authors of either of the original papers. To the best of our knowledge, no antisense transcript of the murine *Sry* gene has been reported. Nevertheless, if the circular version of the *Sry* transcript can soak up miRNAs, can the *Sry* linear transcripts also do the same?

**Figure 1.**
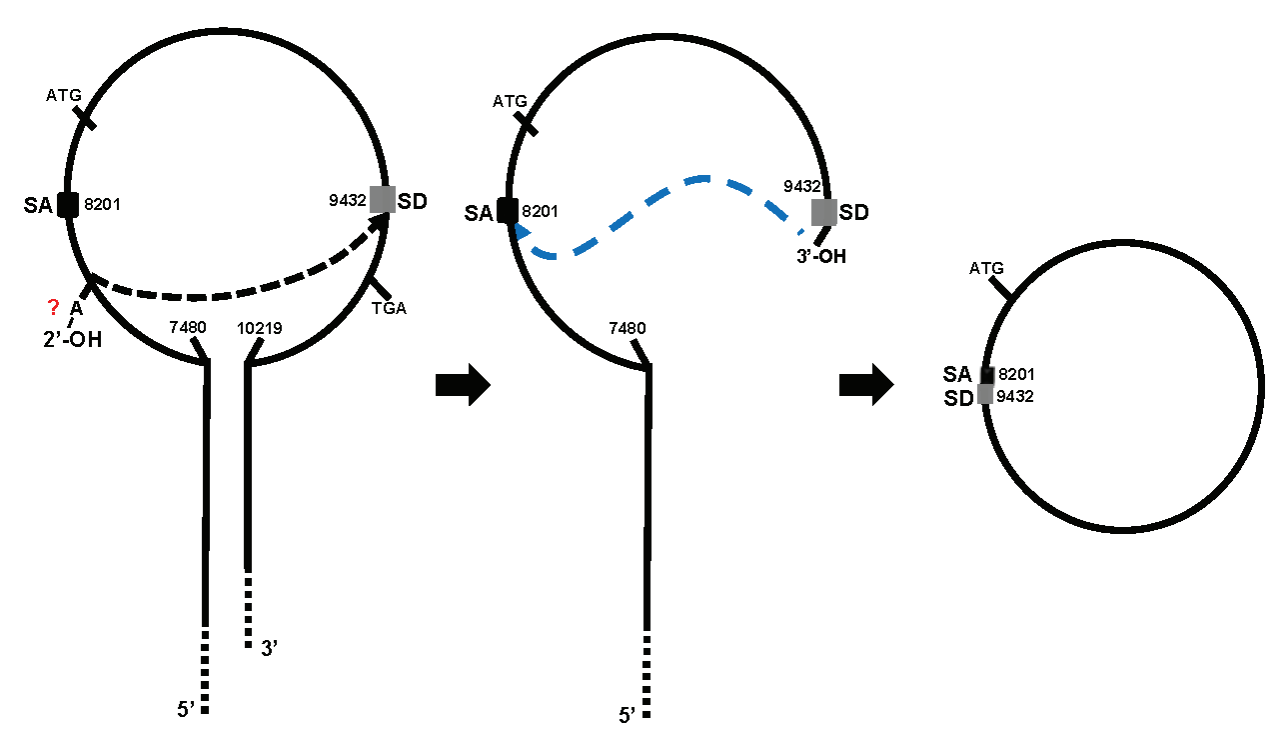
Formation of circular *Sry* RNA After the *Sry* pre-RNA is transcribed, a stem-loop structure is created due to the presence of inverted repeats at the 5′ and 3′ ends. A normal splicing reaction takes place when the splice donor (SD) is attacked by a 2′-OH, presumably from a branch site adenosine residue (A) located in the intron, causing the first cleavage of the phosphodiester backbone. The newly formed 3′-OH at the SD, attacks the 5′-P at the splice acceptor (SA) site, resulting in excision of the intron and ligation of the circular exon of 1231 nucleotides. Modified from Capel *et al.* ^8^.

In this respect, there is evidence that certain miRNAs may function by targeting sites in the 5′-UTR ^15^ and open reading frame (ORF) regions of mRNAs ^16^, suggesting that miRNAs may modulate gene expression by mechanisms different from canonical 3′-UTR target mRNA suppression. Binding of a miRNA to a ceRNA not only prevents that miRNA from binding to other MREs, but can also repress translation from the coding segment of the ceRNA ^17^. A study of the pseudogene of the phosphatase and tensin homolog PTEN, *PTENP1*, provided the first experimental evidence for the cross-talk between coding and non-coding RNAs ^18^. Tay *et al.* found that several endogenous protein-coding transcripts, such as serine incorporator 1 ( *SERINC1*), vesicle-associated membrane protein associated protein A ( *VAPA*), *CCR4-NOT* transcription complex and subunit 6-like ( *CNOT6L*), act as *PTEN* ceRNAs, which regulate PTEN tumor suppressor levels in a miRNA-dependent manner ^18^. This clearly suggests that mRNAs can function as ceRNAs and we propose that the mouse linear *Sry* sense transcript could also behave as a miRNA sponge, or as a ceRNA for miR-138.

Recently, Denzler *et al*. questioned the biological relevance of ceRNAs in terms of the abundance of these molecules which would be required to induce derepression of the targets of specific miRNAs ^19^. However, Memczak *et al.* and Hansen *et al.* shown that circRNA behaving as miRNA sponges selectively bind miRNAs forming complexes with Ago proteins, which raises the possibility that ceRNAs modulate gene expression not only by capturing miRNAs but also through the depletion of the pool of available effector molecules of the miRNA pathway. Additionally, Denzel *et al.* based their calculations for target abundance on sites present in transcriptome 3′ UTRs, however, they were unable to rule out that unidentified highly abundant and regulated non coding RNAs (including circRNAs) might substantially contribute to the pool of available binding sites, a limitation acknowledged in their paper ^19^. This may be of particular importance in the adult testis, which express the circular *Sry* transcript and also has been shown to provide a permissive environment for transcription initiation, a phenomenon that has been called “transcriptional promiscuity” ^20^. Denzel *et al.* also state that their findings in liver tissue can be generalized to other tissue and disease states, given that target abundance did not show large changes in the presence of insulin signaling or liver disease, conditions know to modify gene expression in such tissue. However, the authors also discuss that during cellular processes such as differentiation (like the spermatogenesis in the adult testis), expression of coding and noncoding RNAs changes dramatically, potentially making these systems more amenable to ceRNA-mediated gene regulation ^19^.

Shortly after the emergence of circRNAs, the first public circRNA database ( circBase version 0.1) was developed by the Rajewsky laboratory as a compendium of thousands of circRNAs sequences that are expressed in eukaryotic cells ^21^. Access to this resource allows us to use the information in order to validate those circRNAs that are probably involved in many important cellular processes. Nevertheless, the precise molecular mechanisms that underlie post-transcriptional repression by circRNAs remain still largely unknown, but their discovery demonstrates the importance of this distinct type of non-protein-coding regulatory RNAs for the elucidation of gene function. The extent to which other animal or human antisense or sense circRNAs also behave as miRNA sponges will doubtlessly be a subject of intense research. Moreover, due to their longer half lives *in vivo*, circRNAs may possess a great potential for therapeutic intervention. Thus, manipulating miRNA function, either by mimicking or inhibiting ceRNAs implicated in several disorders such as cancer, could provide a novel strategy to interfere with disease initiation and/or progression. The antisense modulation of circRNAs/ceRNA→miRNAs→mRNAs→protein regulatory networks could offer ingenious decoy combinations (antisense technology) as well as delivery platforms for concurrently target multiple miRNAs in abnormal or undesired conditions ^22^.
