# Supplementary material for: Does the linear Sry transcript function as a ceRNA for miR-138? The sense of antisense
Source: F1000Res. 2014 Nov 13;3:90. Originally published 2014 Apr 11. [Version 2] doi: 10.12688/f1000research.3872.2 (PMC4288412; doi:10.12688/f1000research.3872.2)
Supplement: Peer review history

# Peer review: Referee response for version 2

**DOI:** 10.5256/f1000research.6097.r7098

**Published:** 2014-12-19

**Authors:** Amy Pasquinelli

**Affiliations:** 1 Division of Biological Sciences, University of California San Diego, La Jolla, CA, USA

The revisions are sufficient.

I have read this submission. I believe that I have an appropriate level of expertise to confirm that it is of an acceptable scientific standard.

# Peer review: Referee response for version 2

**DOI:** 10.5256/f1000research.6097.r6689

**Published:** 2014-11-21

**Authors:** Gordon Carmichael

**Affiliations:** 1 Department of Genetics and Developmental Biology, University of Connecticut Health Center, Farmington, CT, USA

This revised version offers substantial improvements and is generally acceptable. Although the fields of circular RNA and miRNA sponges are popular and active at the moment and papers are appearing frequently, it would help if the authors could include in their citations several recent papers that will be of value to readers of this article.

I have read this submission. I believe that I have an appropriate level of expertise to confirm that it is of an acceptable scientific standard.

## References

[1] LasdaEParkerR: Circular RNAs: diversity of form and function.*RNA*.2014;20(12) : 901829-184210.1261/rna.047126.114Reference source25404635PMC4238349

[2] ZhangXOWangHBZhangYLuXChenLLYangL: Complementary sequence-mediated exon circularization.*Cell*.2014;159(1) : 90134-14710.1016/j.cell.2014.09.001Reference source25242744

[3] LiangDWiluszJE: Short intronic repeat sequences facilitate circular RNA production.*Genes Dev*.2014;28(20) : 902233-224710.1101/gad.251926Reference source25281217PMC4201285

[4] BossonADZamudioJRSharpPA: Endogenous miRNA and Target Concentrations Determine Susceptibility to Potential ceRNA Competition. *Mol Cell*.2014;56(3) : 90347-35910.1016/j.molcel.2014.09.018Reference source25449132PMC5048918

# Peer review: Referee response for version 1

**DOI:** 10.5256/f1000research.4147.r4478

**Published:** 2014-10-24

**Authors:** Amy Pasquinelli

**Affiliations:** 1 Division of Biological Sciences, University of California San Diego, La Jolla, CA, USA

This short commentary points out that both sense and antisense transcripts with miRNA target sites could serve to titrate the miRNAs and, thus, release other target RNAs from repression.  Recently, there has been much interest in these so called competing endogenous RNAs (ceRNAs) as a form of regulating miRNA activity.  While ceRNA activity has been attributed to pseudogenes and transcripts that circularize (circRNAs), theoretically any RNA, including protein coding mRNAs, could serve this function.  The correspondence proposes that the linear Sry sense, as well as the previously reported antisense, transcript could function as sponges for miR-138.  A major criticism of the proposal that ceRNA activity plays a function in regulating miRNA availability for target regulation is the consideration of cellular RNA concentrations.  In many cases, the ceRNAs, even the circRNAs which can be more stable, are expressed at levels far below the miRNA or even its target mRNA.  In fact, after this correspondence by Granados-Riveron & Aquino-Jarquin was originally published in April 2014, the hypothesis that ceRNAs can alter miRNA function *in vivo* was rigorously tested and the conclusion was published in May that most ceRNAs simply are not abundant enough to act as competitive inhibitors of miRNA binding to target mRNAs ( Denzler *et al.*, 2014).  Thus, the authors need to include discussion of this concern over how generally miRNA activity might actually be affected by the presence of any kind of ceRNA, sense, antisense or circular.

I have read this submission. I believe that I have an appropriate level of expertise to confirm that it is of an acceptable scientific standard, however I have significant reservations, as outlined above.

# Response

**Published:** 2014-11-06

**Authors:** Guillermo Aquino-Jarquin

**Affiliations:** Hospital Infantil de México, Federico Gómez, Mexico

**This short commentary points out that both sense and antisense transcripts with miRNA target sites could serve to titrate the miRNAs and, thus, release other target RNAs from repression.  Recently, there has been much interest in these so called competing endogenous RNAs (ceRNAs) as a form of regulating miRNA activity.  While ceRNA activity has been attributed to pseudogenes and transcripts that circularize (circRNAs), theoretically any RNA, including protein coding mRNAs, could serve this function.  The correspondence proposes that the linear Sry sense, as well as the previously reported antisense, transcript could function as sponges for miR-138.  A major criticism of the proposal that ceRNA activity plays a function in regulating miRNA availability for target regulation is the consideration of cellular RNA concentrations.  In many cases, the ceRNAs, even the circRNAs which can be more stable, are expressed at levels far below the miRNA or even its target mRNA.  In fact, after this correspondence by Granados-Riveron & Aquino-Jarquin was originally published in April 2014, the hypothesis that ceRNAs can alter miRNA function in vivo was rigorously tested and the conclusion was published in May that most ceRNAs simply are not abundant enough to act as competitive inhibitors of miRNA binding to target mRNAs (Denzler et al., 2014).  Thus, the authors need to include discussion of this concern over how generally miRNA activity might actually be affected by the presence of any kind of ceRNA, sense, antisense or circular.**

Firstly, we thank Dr Pasquinelli for her insightful comments. Secondly, we would like to clarify that the previously reported circular mouse *Sry* transcript reported by Capel *et al*. [ref-1], now known to behave a sponge for mir-138 [ref-2] ^,^[ref-3], is, in fact, not derived from an antisense transcript but from a sense transcript, whose expression is directed by a promoter which is different to the promoter of the canonical linear *Sry* transcript involved in mammalian sex- determination. To the best of our knowledge, no antisense transcript of the murine *Sry* gene has been reported and a recent search of our own in publicly available EST data supports this assertion.  Actually, one of our motivations for publish this piece of correspondence was to clarify this misconception (that the circular *Sry* RNA is an antisense transcript), which was first asserted in the report by Memczak *et al.* in Nature[ref-3] and later reproduced in a review of the subject by Kartha and Subramanian.[ref-4]

We agree that recent findings by Denzler *et al*. question the biological relevance of ceRNAs in terms of the abundance of these molecules which would be required to induce derepression of the targets of specific miRNAs [ref-5]. However, Memczak *et al.* and Hansen *et al. *shown that miRNA sponges selectively bind miRNAs forming complexes with Ago proteins, which raises the possibility that ceRNAs modulate gene expression not only by capturing miRNAs but also through the depletion of the pool of available effector molecules of the miRNA pathway. Additionally, Denzel *et al.* based their calculations for target abundance on sites present in transcriptome 3´UTRs, however, they were unable to rule out that unidentified highly abundant and regulated non coding RNAs (including circRNAs) might substantially contribute to the pool of available binding sites, a limitation acknowledged in their paper [ref-5]. This may be of particular importance in the adult testis, which express the circular *Sry* transcript and also has been shown to provide a permissive environment for transcription initiation, a phenomenon that has been called "transcriptional promiscuity" [ref-6].  Denzel *et al.* also state that their findings in liver can be generalized to other tissues and disease states, given that target abundance did not show large changes in the presence of insulin signaling or liver disease, conditions know to modify gene expression in hepatocytes.  However, the authors also discuss that during cellular processes such as differentiation (like the spermatogenesis in the adult testis), expression of coding and non-coding RNAs changes dramatically, potentially making these systems more amenable to ceRNA-mediated gene regulation [ref-5]. 

***We agree with Dr Pasquinelli in respect to the need of a discussion on the proposed mechanisms for ceRNAs action and their caveats in light of the findings by Denzel et al. and therefore, the new version of our correspondence includes such discussion.***

[References]

[[1|type=journal|title=Circular transcripts of the testis-determining gene Sry in adult mouse testis|authors=Capel/B;Swain/A;Nicolis/S;Hacker/A;Walter/M;Koopman/P;Goodfellow/P;Lovell-Badge/R|source=Cell|year=1993|vol=73|issue=5|fpage=1019|lpage=1030|pmid=7684656|doi=10.1016/0092-8674(93)90279-Y|url=http://www.cell.com/cell/abstract/0092-8674(93)90279-Y]]

[[2|type=journal|title=Natural RNA circles function as efficient microRNA sponges|authors=Hansen/TB;Jensen/TI;Clausen/BH;Bramsen/JB;Finsen/B;Damgaard/CK;Kjems/J|source=Nature|year=2013|vol=495|issue=7441|fpage=384|lpage=388|pmid=23446346|doi=10.1038/nature11993|url=http://www.nature.com/nature/journal/v495/n7441/full/nature11993.html]]

[[3|type=journal|title=Circular RNAs are a large class of animal RNAs with regulatory potency|authors=Memczak/S;Jens/M;Elefsinioti/A;Torti/F;Krueger/J;Rybak/A;Maier/L;Mackowiak/SD;Gergersen/LH;Munschauer/M;Loewer/A;Ziebold/U;Landthaler/M;Kocks/C;le Noble/F;Rajewsky/N|source=Nature|year=2013|vol=495|issue=7441|fpage=333|lpage=338|pmid=23446348|doi=10.1038/nature11928|url=http://www.nature.com/nature/journal/v495/n7441/full/nature11928.html]]

[[4|type=journal|title=Competing endogenous RNAs (ceRNAs): new entrants to the intricacies of gene regulation|authors=Kartha/RV;Subramanian/S|source=Front Genet|year=2014|vol=5|fpage=8|pmid=24523727|pmcid=3906566|doi=10.3389/fgene.2014.00008|url=http://journal.frontiersin.org/Journal/10.3389/fgene.2014.00008/full]]

[[5|type=journal|title=Assessing the ceRNA hypothesis with quantitative measurements of miRNA and target abundance|authors=Denzler/R;Agarwal/V;Stefano/J;Bartel/DP;Stoffel/M|source=Mol Cell|year=2014|vol=54|issue=5|fpage=766|lpage=776|pmid=24793693|doi=10.1016/j.molcel.2014.03.045|url=http://www.cell.com/molecular-cell/abstract/S1097-2765(14)00278-0]]

[[6|type=journal|title=Transcriptional promiscuity in testes|authors=Schmidt/EE|source=Curr Biol|year=1996|vol=6|issue=7|fpage=768|lpage=769|pmid=8805310|doi=10.1016/S0960-9822(02)00589-4|url=http://www.cell.com/current-biology/abstract/S0960-9822(02)00589-4]]

# Peer review: Referee response for version 1

**DOI:** 10.5256/f1000research.4147.r5178

**Published:** 2014-06-19

**Authors:** Gordon Carmichael

**Affiliations:** 1 Department of Genetics and Developmental Biology, University of Connecticut Health Center, Farmington, CT, USA

This article presents a short summary and review of recent work on circular RNAs and on competing endogenous RNAs, which can sequester and “sponge” microRNAs.  A point made by the authors is that both sense and antisense RNAs from the same genomic region can act as miRNA sponges and that linear transcripts from the same regions that produce circular RNAs can themselves sequester miRNAs.  Overall, I found the article to be provocative, though not novel.  During the past several years there have appeared a number of papers describing circular RNAs of various sorts (for example, stable introns and circles resulting from splicing events commonly promoted by strong secondary structures flanking exons).  There has also been a wealth of published work suggesting or showing that abundant RNAs can bind miRNAs and thus reduce their availability for the regulation of some mRNAs.  Thus, this paper breaks little new ground, though it does point out that many genomic regions express both sense and antisense transcripts, and each of these can affect miRNA availability.  To me, the area covered by this paper is a very interesting and important one, and it is of value to present and discuss new concepts in gene regulation. Thus, I think this manuscript would be improved by:

1. More clearly summarizing the ceRNA field.
2. Discussing a bit more the well known fact that sense and antisense transcripts are commonly expressed in cells, though not necessarily from the same locus at the same time.
3. Mentioning the several ways that stable circular RNAs can be produced.
4. Finally, discussing the issue lacking from the current version that, in order to effectively act as a miRNA sponge, an RNA must not only be stable, but also in the proper cellular compartment and containing a molar concentration of miRNA binding sites that is high enough to sequester a biologically significant fraction of the endogenous miRNA of interest.  

I have read this submission. I believe that I have an appropriate level of expertise to confirm that it is of an acceptable scientific standard, however I have significant reservations, as outlined above.

# Response

**Published:** 2014-11-06

**Authors:** Guillermo Aquino-Jarquin

**Affiliations:** Hospital Infantil de México, Federico Gómez, Mexico

**1. More clearly summarizing the ceRNA field.**

We are grateful to Dr. Gordon Carmichael for all the comments made to our manuscript.

A recently discovered molecular mechanism, named Competing Endogenous RNA (ceRNA) effect, has highlighted the importance of indirect interactions among transcript RNAs competing for the same pool of miRNAs [ref-1]. ceRNAs share one or more miRNA response elements (MREs) and compete for a restricted pool of common miRNAs. Thus, ceRNAs form complex regulatory networks of miRNAs and MRE-containing transcripts, both protein-coding and noncoding, which ensure a tight control of many biological processes. Aberrant expression of central nodes of such ceRNA networks may cause a disturbance that could contribute to disease pathogenesis [ref-2]. A discussion along these lines has been included in our new submission.

**2. Discussing a bit more the well known fact that sense and antisense transcripts are commonly expressed in cells, though not necessarily from the same locus at the same time.**

*CDR1as* is a circRNA that functions as a sponge for miR-7, deriving from an antisense transcript of the CDR1 protein-coding gene. On the other hand, the transcript of the male sex-determining gene *Sry*, transcribed in sense orientation [ref-3], is a second circRNA proposed to act as a sponge, in this case, for miR-138. However, Memczak *et al.* stated that “Perhaps the best known circRNA is **antisense** to the mRNA transcribed from the *SRY* (sex-determining region Y) locus and is highly expressed in testes” [ref-4]. Recently, Kartha and Subramanian asserted, based on the report by Hansen *et al.*, that this *Sry *RNA is an antisense circular transcript that functions as a miRNAs sponge [ref-5]. Although this apparently is a typographical error (antisense instead of sense), it was also referred as such in the original report by Memczak *et al.* in Nature[ref-4]. This would suggest that the circular *Sry* transcript is, as occurs with the CDR1as sponge, an antisense circular RNA. Although it seems obvious that sponges are antisense to the miRNA they bind to, it should not be assumed that all circRNAs are transcripts in an antisense orientation to a protein coding gene, as occurs with CDR1as. We conducted an *in silico* analysis taking the antisense sequence of the *Sry* mRNA to make a search for potential binding sites for miR-138, using the fuzzy bioinformatics tool (EMBOSS Suite) and we found none. When we used the *Sry* ORF (GenBank NM_011564) for the miR-138 binding site (CACCAGCA), we found 14 potential sites which is consistent with the number of sites found by Hansen and colleagues. For this reason, we remark in our current submission that both sense and antisense Sry transcripts could circularize and behave as miRNAs sponges, and importantly, that protein-coding segments of *Sry *mRNA could also assume this role.

To our knowledge, there are no reports of a circRNA whose sequence in sense and antisense orientation possesses the ability to function as miRNA sponge. On the other hand, a particularly interesting family of non-protein coding RNAs consists of natural antisense transcripts (NATs). NATs are ncRNAs transcribed from the opposite strand of a coding gene and are capable of regulating the expression of their sense gene pair or of several related genes [ref-6]. Genomic loci that express NATs are highly abundant and sense/antisense (SAS) transcript pairs tend to be co-expressed. The most comprehensive studies predict that in human and mice 40-72% of all transcriptional units show evidence of bi-directional transcription [ref-7]. The regulatory activity of SAS pairs in human tissues has been postulated on protein expression at different levels, such as alternative splicing, post-transcriptional regulation, transport and epigenetic imprinting as well as transcriptional and translational interference through annealing to complementary sequences [ref-8]. ***As requested, we have included this information in the version of our submission.***

**3. Mentioning the several ways that stable circular RNAs can be produced.** 

Recent bioinformatic and experimental analyses have identified thou­sands of circRNAs in the mammalian transcriptome, suggesting that circRNAs may in fact represent a new class of ceRNA regulators [ref-9]. These circRNAs are produced mainly through a type of alternative RNA splicing named ‘back-splicing’, in which a splice donor splices to an upstream acceptor rather than a downstream acceptor [ref-10]. Recently Guo *et al.* suggested that this would be the way in which generates most, if not all, cellular circRNAs *. **Please find a relevant discussion in the new version.***

**4. Finally, discussing the issue lacking from the current version that, in order to effectively act as a miRNA sponge, an RNA must not only be stable, but also in the proper cellular compartment and containing a molar concentration of miRNA binding sites that is high enough to sequester a biologically significant fraction of the endogenous miRNA of interest.  ** 

Increasing experimental evidence supports the hypothesis that mul­tiple non-coding RNA species, including small non-coding RNAs, pseudogenes, lncRNAs and circular RNAs (circRNAs) may pos­sess ceRNA activity [ref-9]. The effectiveness of a ceRNA would depend on the number of miRNAs that it can ‘‘absorb’’ This, in turn, would depend on the ceRNA’s accessibility to miRNA molecules, which is influenced by its subcellular localization and its interaction with RNA-binding proteins. Furthermore, the specific cellular context in which the ceRNA is expressed would also impact its overall influence because not all microRNAs are present ubiquitously and at all times [ref-11]. ***We have included a discussion of the subject in the current version.***

[References]

[[1|type=journal|title=Modelling Competing Endogenous RNA Networks|authors=Bosia/C;Pagnani/A;Zecchina/R|source=PLoS One|year=2013|vol=8|issue=6|pmid=23840508|pmcid=3694070|doi=10.1371/journal.pone.0066609|url=http://www.plosone.org/article/info%3Adoi%2F10.1371%2Fjournal.pone.0066609]]

[[2|type=journal|title=Pseudogenes as competitive endogenous RNAs: target prediction and validation|authors=Karreth/FA;Ala/U;Provero/P;Pandolfi/PP|source=Methods Mol Biol|year=2014|vol=1167|fpage=199|lpage=212|pmid=24823779|doi=10.1007/978-1-4939-0835-6_13|url=http://link.springer.com/protocol/10.1007%2F978-1-4939-0835-6_13]]

[[3|type=journal|title=Circular transcripts of the testis-determining gene Sry in adult mouse testis|authors=Capel/B;Swain/A;Nicolis/S;Hacker/A;Walter/M;Koopman/P;Goodfellow/P;Lovell-Badge/R|source=Cell|year=1993|vol=73|issue=5|fpage=1019|lpage=1030|pmid=7684656|doi=10.1016/0092-8674(93)90279-Y|url=http://www.cell.com/cell/abstract/0092-8674(93)90279-Y]]

[[4|type=journal|title=Circular RNAs are a large class of animal RNAs with regulatory potency|authors=Memczak/S;Jens/M;Elefsinioti/A;Torti/F;Krueger/J;Rybak/A;Maier/L;Mackowiak/SD;Gregersen/LH;Munschauer/M;Loewer/A;Ziebold/U;Landthaler/M;Kocks/C;le Noble/F;Rajewsky/N|source=Nature|year=2013|vol=495|issue=7441|fpage=333|lpage=338|pmid=23446348|doi=10.1038/nature11928|url=http://www.nature.com/nature/journal/v495/n7441/full/nature11928.html]]

[[5|type=journal|title=Competing endogenous RNAs (ceRNAs): new entrants to the intricacies of gene regulation|authors=Kartha/RV;Subramanian/S|source=Front Genet|year=2014|vol=5|issue=8|pmid=24523727|pmcid=3906566|doi=10.3389/fgene.2014.00008|url=http://journal.frontiersin.org/Journal/10.3389/fgene.2014.00008/full]]

[[6|type=journal|title=Natural antisense transcripts|authors=Khorkova/O;Myers/AJ;Hsiao/J;Wahlestedt/C|source=Hum Mol Genet|year=2014|vol=23|issue=R1|fpage=R54|lpage=R63|pmid=24838284|pmcid=4170719|doi=10.1093/hmg/ddu207|url=http://hmg.oxfordjournals.org/content/23/R1/R54.long]]

[[7|type=journal|title=Contribution of natural antisense transcription to an endogenous siRNA signature in human cells|authors=Werner/A;Cockell/S;Falconer/J;Carlile/M;Alnumeir/S;Robinson/J|source=BMC Genomics|year=2014|vol=15|issue=19|pmid=24410956|pmcid=3898206|doi=10.1186/1471-2164-15-19|url=http://www.biomedcentral.com/1471-2164/15/19]]

[[8|type=journal|title=Genome-wide natural antisense transcription: coupling its regulation to its different regulatory mechanisms|authors=Lapidot/M;Pilpel/Y|source=EMBO Rep|year=2006|vol=7|issue=12|fpage=1216|lpage=1222|pmid=17139297|pmcid=1794690|doi=10.1038/sj.embor.7400857|url=http://embor.embopress.org/content/7/12/1216.long]]

[[9|type=journal|title=The multilayered complexity of ceRNA crosstalk and competition|authors=Tay/Y;Rinn/J;Pandolfi/PP|source=Nature|year=2014|vol=505|issue=7483|fpage=344|lpage=352|pmid=24429633|pmcid=4113481|doi=10.1038/nature12986|url=http://www.nature.com/nature/journal/v505/n7483/full/nature12986.html]]

[[10|type=journal|title=Expanded identification and characterization of mammalian circular RNAs|authors=Guo/JU;Agarwal/V;Guo/H;Vartel/DP|source=Genome Biol|year=2014|vol=15|issue=7|fpage=409|pmid=25070500|pmcid=PMC4165365|doi=10.1186/s13059-014-0409-z|url=http://genomebiology.com/content/15/7/409]]

[[11|type=journal|title=A ceRNA hypothesis: the Rosetta Stone of a hidden RNA language?|authors=Salmena/L;Poliseno/L;Tay/Y;Kats/L;Pandolfi/PP|source=Cell|year=2011|vol=146|issue=3|fpage=353|lpage=358|pmid=21802130|pmcid=3235919|doi=10.1016/j.cell.2011.07.014]]
